# Should young athletes be screened for cardiomyopathies to reduce the burden of sudden cardiac death in athletes?

**DOI:** 10.1007/s12551-023-01085-2

**Published:** 2023-06-23

**Authors:** Grace McColgan, Mauricio Villarroel, Katja Gehmlich

**Affiliations:** 1 College of Medical and Dental Sciences, University of Birmingham, Edgbaston, Birmingham, B15 2TT UK; 2 Institute of Biomedical Engineering, Department of Engineering Science, University of Oxford, Oxford, OX3 7DQ UK; 3 Institute of Cardiovascular Science, University of Birmingham, Edgbaston, Birmingham, B15 2TT UK; 4 Division of Cardiovascular Medicine, Radcliffe Department of Medicine, and British Heart Foundation Centre of Research Excellence Oxford, University of Oxford, Oxford, OX3 9DU UK

**Keywords:** Hypertrophic cardiomyopathy, Arrhythmogenic cardiomyopathy, Arrhythmia, Sudden cardiac death, Electrocardiogram, Machine learning, Wearables

## Abstract

In this correspondence, we highlight the risk of sudden cardiac death associated with undiagnosed cardiomyopathies. Life-threatening arrhythmias, which underlie sudden cardiac death, can be triggered by high-intensity exercise. It raises the question whether, and if so, how athletes should be screened for cardiomyopathies. The example of practice from Italy is discussed. We also briefly discuss novel developments, such as wearable biosensors and machine learning, which could be applied to screening for cardiomyopathies in future.

## Introduction

Sudden cardiac death (SCD) is commonly defined as death from the loss of cardiac function, occurring unexpectedly within 1 h (or within 24 h in unwitnessed cases) from the onset of symptoms (Han et al. 2023). SCD can occur in apparently healthy individuals, including professional athletes, causing arrhythmic events such as ventricular tachycardia or fibrillation. Fabrice Muamba, a professional football (soccer) player, collapsed on the pitch during a Football Association cup match on March 17th, 2012. His heart stopped for 78 min, all while helpless fans watched him being resuscitated on the pitch. This was not an isolated event; Antonio Puerta and Marc-Vivien Foé, both also professional football players, experienced similar life-threatening arrhythmic events on the pitch (Higgins and Andino 2013).

The epidemiology and aetiology of SCD in athletes are an active research area (Harmon 2022). The most common cause of SCD in athletes under the age of 35 is cardiomyopathies (Maron et al. 1996), particularly Hypertrophic and Arrhythmogenic Cardiomyopathy. The diagnosis and prevention of SCD in young athletes remain a difficult medical challenge (Modesti et al. 2022). In this correspondence, we explore the question whether athletes should be screened for SCD and related arrhythmic events as they progress through their athletic careers. We discuss solutions that maximise the protection of athletes’ health while also being practical and sustainable in the long term.

## Cardiomyopathies

Cardiomyopathy is defined as an anatomic and pathologic diagnosis associated with muscular or electrical dysfunction of the heart (Chandra et al. 2013). This dysfunction can be acquired or inherited (i.e. genetic). Cardiomyopathies can initially be asymptomatic but may progress over time to heart-failure-like symptoms such as dyspnoea and oedema (Chandra et al. 2013). The two main inherited arrhythmogenic cardiomyopathies at risk of SCD are Hypertrophic and Arrhythmogenic cardiomyopathy (Watkins et al. 2011).

Hypertrophic Cardiomyopathy (HCM) is an autosomal dominant condition that affects 1 in 500 people (Maron et al. 1995). It is characterised by the thickening of the left ventricular wall in the absence of increased external load. Molecular hallmarks are myocyte disarray or cardiac fibrosis, which lead to increased end-diastolic pressure; symptoms of heart failure; and cardiac arrhythmias such as atrial fibrillation and non-sustained-ventricular-tachycardia (Alcalai et al. 2008). HCM genetic studies have revealed over 400 mutations that affect > 10 genes that encode proteins of the cardiac sarcomere (Marian 2021). The most common ones are genetic variants in *MYH7* and *MYBPC3* genes, both encoding proteins of the thick filament (Alcalai et al. 2008). Changes in calcium handling are thought to make individual cardiomyocytes proarrhythmic, which, alongside with the fibrous tissue replacement, can cause re-entrance arrhythmias (Coppini et al. 2018).

Arrhythmogenic Cardiomyopathy (ACM) is most commonly autosomal dominant, with a prevalence of 1:1000 to 1:5000 (Corrado et al. 2020). The pathological hallmarks of this condition are myocardial atrophy and fibrofatty replacement (Corrado et al. 2020). The most commonly affected genes in ACM code for desmosomal proteins such as Desmoplakin (*DSP*), plakophilin (*PKP2)*, Desmocollin (*DSC2*) and Desmoglein (*DSG2*) (El Hadi et al. 2023). Desmosomes are cell–cell contact structures that form part of the intercalated disc and play a key role in cellular integration, intercellular signalling and electrochemical coupling between the cardiac myocytes (El Hadi et al. 2023). The latter is thought to result in arrhythmia. In young adults, ACM presents clinically with palpitations, presyncope, syncope, ventricular tachycardias and unfortunately sudden cardiac death. ACM is the most common cause of SCD in young athletes in Italy (Corrado et al. 2003).

## Aim of screening

Screening can be defined as the systematic testing of asymptomatic individuals for a preclinical disease. The purpose is to prevent or delay the development of advanced disease through early diagnosis and treatment (Morrison 1992). In the case of HCM and ACM in young competitive athletes, the focus would be identifying young athletes with cardiomyopathies who would have an elevated risk of SCD. Preventative measures to reduce their risk can be provided, or alternatively, a ban could be placed upon the athlete from participating in competitive sports. When arguing in favour of screening for cardiomyopathies, there are two important factors to consider: the effectiveness of the screening programme in identifying the condition and the potential effectiveness of the intervention following the diagnosis.

## Effectiveness of ECG screening

A key case study for the effectiveness of identifying cardiomyopathies through screening is the programme currently in place in Italy. Pre-participation screening is mandatory for athletes between 12 and 35 years of age who are engaged in organised and competitive sports. It has even been hypothesised to be the reason behind the reduced frequency of SCD due to HCM in Italy (Corrado et al. 2008; Pelliccia and Maron 1995). In Italy, the national pre-participation screening process involves a medical history, physical examination and a 12-lead ECG recording which aims to identify asymptotic athletes with HCM (Corrado et al. 2008). In a cohort study, Antonio Pelliccia et al. (1995) investigated the effectiveness of the Italian national pre-participation programme of HCM in competitive athletes at identifying and disqualifying athletes who had HCM. The study included 4450 athletes who were considered to compete at either a national or an international level and had been identified as elite athletes. Approximately 2–8 months following initial participation in the national screening programme where the athletes were considered ‘cleared’, all the 4450 athletes were invited to have a further evaluation involving two-dimensional and Doppler echocardiography. In athletes where abnormalities were found, but case diagnosis could not be definite, other tests were performed such as cardiac magnetic resonance imaging or coronary angiography. In addition, genetic testing was carried out via direct Sanger DNA sequencing for the most common HCM disease genes (Alcalai et al. 2008). As multiple techniques had to be used to reach a definite diagnosis, it raises the question if an ‘ideal’ screening programme needs to have multiple diagnostic techniques.

For HCM diagnosis to be suspected, LV wall thickness was to be greater than 13 mm on echocardiogram without the presence of another cardiac systemic disease that would cause such hypertrophy. The results showed that out of the 4450 athletes, 4397 had HCM diagnosis excluded based on normal LV wall thickness. Twelve athletes had other cardiovascular diseases such as ACM, aortic valve disease and myocarditis. The remaining 41 athletes displayed left ventricular hypertrophy and underwent the further testing previously detailed. Thirty of these athletes had increased LV wall thickness (13–15 mm) accompanied by distinct cavity enlargement. This led the researchers to conclude they did not have HCM but physiological hypertrophy and cardiac remodelling, which is characteristic of athlete’s heart (Prior and La Gerche 2012). This raises the question of whether measuring LV wall thickness can be adequate for screening cardiomyopathy when athlete’s heart is a confounding condition with similar expression; this will be explored later in this evaluation. Lastly, 4 athletes were categorised in the ‘grey zone’, which is where LV thickness was 13 mm and marked non-dilated LV cavity was present, indicating that the diagnosis would not be athlete’s heart. Over an 8–12-year period, the 4 athletes were followed up with several echocardiograms. Three different diagnoses were made: One athlete had increased wall thickness during the follow-up period and underwent further genetic testing. The new test identified a *MYPBC3* genetic variant (E542Q) that had previously been described as associated with HCM (Alcalai et al. 2008). Therefore, the athlete was given a definite diagnosis of HCM. A second athlete started to experience episodes of non-sustained-ventricular-tachycardia along with a borderline LV thickness of 13 mm. The athlete was classified as a potential HCM case. Both athletes were removed from training and competitions, in line with the current Italian guidelines. The two remaining athletes were diagnosed with non-pathological cardiac conditions; no restrictions were placed on their athletic careers.

If the Italian screening programme had no effect on identifying HCM at all, any individual with HCM would have remained in the cohort. Statistically, this would affect five to ten athletes, who would be predicted to be diagnosed with HCM in the further, more in-depth testing conducted. However, as only one definite and one potential case were identified, the team concluded that the screening programme was effective—more specifically, that the 12-lead ECG is an effective tool for identifying HCM and that echocardiography is not necessary for large-scale-screening programmes. This highlights that ECGs would be useful for screening programmes worldwide, and given that they are more cost-effective and easier to apply than echocardiography, they may be more sustainable and cost-effective in areas with a smaller budget for screening.

However, the main critique of this study, and of the Italian screening programme, is its focus on only HCM. They did not equally consider ACM, even though it has been shown to pick up ACM cases in later studies (Cicenia et al. 2022). Moreover, the athletes who have been diagnosed with HCM were disqualified from all sporting events. This is a questionable aspect of the Italian screening programme as this sudden expulsion can cause major emotional distress for the athletes (Wexler et al. 2009). It sparks the question which treatments can be offered so that athletes with cardiomyopathies can continue with sporting activities.

## Cardioverter defibrillators

Building on from the criticisms of the previous study (Pelliccia and Maron 1995), it is important to assess the potential effectiveness of the interventions following a diagnosis of cardiomyopathy. One potential option could be Implantable Cardioverter Defibrillators (ICD). They are a form of primary prevention where a small device is implanted to continuously monitor heart electrical activity. It can deliver electrical pulses when dangerous abnormal heart rhythms occur, such as ventricular fibrillation, and thereby prevent SCD (DiMarco 2003). Currently, the American Heart Association and the European Society of Cardiology agree that patients who have an ICD fitted should be restricted to moderate, leisure-time physical activity such as bowling and cricket (Zipes et al. 2005). However, despite this recommendation, many athletes who have an ICD implanted may wish to continue physical activity, as permanent exclusion can cause major psychological impacts such as depression (Lavallee et al. 1997). The main concern is that vigorous physical exercise can cause inappropriate shocks and damage to the ICD that would result in failure to prevent life-threatening arrhythmias and therefore cardiac death. However, no athletic deaths were recorded in nearly 400 athletes with ICDs in over 3 years of follow-up (Ponamgi et al. 2015).

Further studies are needed to confirm whether vigorous exercise is safe for athletes with ICDs. Currently, the consensus is ‘shared decision’. The physician communicates the risks and benefits to the patient and often their family as well, to help them reconcile the options available with their personal preferences and values (Lampert 2019). This patient-centred approach towards ICD implantation in athletes offers a potential solution for avoiding complete exclusions from sports. However, it requires clear communication, willingness to engage the patients and ability of the patients to understand the complex implications of their decisions.

As an alternative to ICDs, automated external defibrillators are devices that can detect arrhythmias and provide shocks to rest cardiac rhythm. They can be used on any individual suspected to have a life-threatening cardiac arrhythmia and are designed to be used by untrained personnel. Making automated external defibrillators widely available at all competitive sporting events could prevent sudden cardiac death events (Garg 2015).

## Challenges of screening

Having evaluated the argument in favour of screening athletes for cardiomyopathy, it is important to evaluate the challenges that are currently preventing global roll-out of such screening programmes.

### False positives rates

One area of key interest is the incidence and impacts of false positives, i.e. when a person is diagnosed with a condition they do not have. It can have serious ramifications, e.g. in the case of athletes’ screening programmes, it can lead to permanent disqualification from their sport.

The most likely false positive diagnosis in athletes’ screening is athlete’s heart being mis-diagnosed as HCM (Pelliccia et al. 2000). Athlete’s heart, also known as exercise-induced adaptive myocardial hypertrophy, is the term given to the complex of structural, functional and electrical remodelling caused by regular athletic training. This physiological response has been well established for long-distance athletes, e.g. marathon runners and triathletes (Franzen et al. 2013). The hallmark of athlete’s heart is hypertrophy of the left ventricle due to exercise (Prior and La Gerche 2012). The increased haemodynamic load initiates the hypertrophic response where cardiomyocytes synthesise new contractile proteins and assemble new sarcomere units in parallel. This causes the cardiomyocytes to expand resulting in increased wall thickness (Mihl et al. 2008). The similar presentation to HCM, which is also characterised by LV hypertrophy (Alcalai et al. 2008), provides a challenge to design a screening programme for HCM based on LV wall thickness. Some athletes will have non-pathological LV thickness due to their athletic training; thus, some individuals will fall into a ‘grey zone’ of elevated LV wall thickness (Pelliccia and Maron 1995).

Pelliccia et al. (2000) analysed the prevalence, clinical significance and determinates of abnormal ECG findings. Their aim was to clarify abnormal ECG recordings seen in trained athletes, such as increased QRS voltage, which can be suggestive of left ventricular hypertrophy (Pelliccia et al. 2000). The Institute of Sports Science of the Italian national Olympic committee evaluated ECG recordings from 1005 athletes without knowledge of the clinical history or the echocardiographic findings of the athletes. Three categories were created from the athlete’s ECG recordings: distinctly abnormal, mildly abnormal or normal. In the distinctly abnormal category were athletes whose ECG recordings were strongly indicative of cardiovascular disease. The mildly abnormal category contained cases potentially indicative for the presence of cardiovascular disease. The group with ‘normal’ ECG recordings contained only minor alterations, ruled out as athlete’s heart. Out of the 1005 athletes, 402 (40%) presented with abnormal ECG recordings with 145 falling in the distinctly abnormal category and 257 falling into the mildly abnormal category. Of the remaining 603 athletes, 188 had completely normal ECG and 415 had minor alterations consistent with athlete’s heart. When looking at the athletes with cardiovascular abnormalities, 27 had abnormal ECG recordings and 26 had normal ECG recordings (false negatives). On the other hand, of the 952 athletes without cardiovascular abnormalities, 577 had normal ECGs and 375 had mildly or distinctly abnormal ECGs. This means the categories of mildly and distinctly abnormal ECG recordings had a positive predictive accuracy of 7% and a negative predictive accuracy of 96% for detecting cardiovascular abnormalities. When reviewing the positive predictive accuracy, out of all the athletes being categorised as having abnormal ECGs, only 7% will have a cardiovascular abnormality. A specific subgroup was identified by the study; 145 athletes had abnormal ECG patterns that had indicated a diagnosis of cardiovascular disease even though only a small minority had clinical or echocardiographic evidence of this. The majority of these athletes had increased LV dimensions that would explain the abnormal ECG, which is a common feature of athlete’s heart syndrome. This study has therefore highlighted that in 40% of the athletes who had abnormal ECGs, the majority of them were just indicative of cardiac remodelling indicative of athlete’s heart.

In contrast to a previous study (Pelliccia and Maron 1995), the authors here conclude that ECG recordings cannot distinguish physiological cardiac remodelling from HCM (Pelliccia et al. 2000). This would increase the number of athletes that unnecessarily undergo further testing. Based on estimates for the USA, the costs for further testing can include $104 for an exercise test, $431 for an echocardiogram and $160 for a Holter monitor (Hill et al. 2011). If 10.7 million participants under the ages of 40 went through the pre-screening programme, the cost of unnecessary tests would be over $1 billion (Hill et al. 2011). While these costs do not consider the amount saved by early detection of cardiomyopathy, it raises the question of whether ECG screening is the most cost-effective method for national screening programmes.

### ECG interpretation

One possible reason for the incidences of false positives could be that the physicians involved are not adequately trained to interpret the ECG findings accurately. A 2011 study aimed to evaluate the accuracy of paediatric cardiologists’ interpretations of ECG recordings (Hill et al. 2011). Fifty three members of the Western Society of Paediatric Cardiology Society were asked to interpret a set of ECG recordings with various cardiovascular abnormalities and asked to identify them.

Out of 212 cases to diagnose HCM, it was only correctly identified 128 times (success rate of 60%). For 40% of the time, the physicians would have caused false negative diagnoses where a patient is incorrectly told they do not have a condition. This would create a false sense of security for the patient and could have large ramifications such as lack of intervention, increasing the risk of SCD. This study highlights that there is a need for extra training to enhance physicians’ ability to interpret abnormal ECG findings and reduce the number of referrals to additional services such as echocardiograms to confirm diagnoses. This would also increase the cost-effectiveness of the programme. The major downside of this, however, is that additional training requires time and resources, unless automated algorithms can be employed.

## Future directions

A potential area of interest for further development of the screening programmes and cardiomyopathy detection are wearable biosensors (often called ‘wearables’). These are already commonly used by athletes and active individuals to monitor heart rate and other physiological parameters. A review from 2021 suggests that wearables could play a crucial role in risk stratification and prevention of SCD through the identification of arrhythmias (Rao et al. 2021). The ability of wearables to detect arrhythmias was highlighted by the Apple Heart Study Test (Perez et al. 2019). This study virtually enrolled 419,297 participants within 8 months. A photoplethysmography-based algorithm detected an irregular pulse in 0.52% of the individuals. The positive predictive value for the irregular pulse notification was 84%. While this study was focused on atrial fibrillation, it highlights that there is potential for wearable devices to detect arrhythmias, which are seen in both HCM and AVRC.

Another exciting field of development is the application of machine learning algorithms to automated pattern recognition of ECGs, which may help to better risk stratify individuals for cardiomyopathy based on ECG recording (Minchole et al. 2019). This has been applied to the risk prediction of arrhythmias in HCM patients (Lyon et al. 2018a, b; Lyon et al. 2018a, b). A recent study suggested that an automated algorithm had a higher sensitivity of automated HCM detection than three specialists (Goto et al. 2022). Future work will need to test whether such automated algorithms are able to distinguish ECG features of cardiomyopathy from Athlete’s heart.

In parallel, there are advancements in the field of genetic screening, i.e. more individuals will have genomes/exomes interrogated and incidental findings could theoretically help to assign risk for cardiomyopathies. However, in reality, most incidental findings are likely to be considered variant of unknown significance (VUS) and are unlikely to be actionable without further clinical evaluation (Hershberger et al. 2018). A recent collection of case reports (Ezekian et al. 2021) highlights the dilemma of VUS in incidental genetic findings and emphasises the importance of pre-test probability of the condition and signal to noise ratio of the genetic testing when interpreting incidental findings. The authors further suggest an algorithm for the interpretation of incidental variants found in genes associated with cardiomyopathy, which could also be applied to incidental genetic findings in athletes.

## Conclusions

This correspondence evaluated the positives and negatives factors of implementing a screening programme for cardiomyopathies. A screening programme has the potential to be effective at identifying cardiomyopathies and helping reduce the incidence of SCD in athletes. The current recommendations of ECG screening and disqualification are not adequate for worldwide implementation. There is a need to allocate resources to improve on physician training programmes to reduce the false positive incidence for ECG interpretation. Additionally, new alternatives to athletes being disqualified from sports activity need to be proposed to reduce the associated emotional distress.

Novel technological developments, such as machine learning algorithms and wearables, might support the development of efficient, less invasive screening programmes in future, which depend less on specialists to, be carried out.
